# Morphometric vertebral fracture risk in women with postsurgical hypoparathyroidism

**DOI:** 10.3389/fendo.2022.948435

**Published:** 2022-12-23

**Authors:** Catarina Brasil d’Alva, André Rodrigues Façanha Barreto, Marcello H. Nogueira-Barbosa, Caio César Cavalcante Arruda, Áurea Maíla Albuquerque, Débora Mota Cordeiro Praciano, Rodrigo Ponte Viana, Daniel Duarte Gadelha, Antônio Brazil Viana Jr, Ilana Marques Moreira, Francisco José Rodrigues de Moura Filho, Ana Flávia Torquato de Araújo Junqueira, Renan Magalhães Montenegro Jr

**Affiliations:** ^1^ Núcleo de Atendimento Multidisciplinar às Doenças Osteometabólicas, Depto. de Medicina Clínica da Universidade Federal do Ceara, Fortaleza, Brazil; ^2^ Serviço de Radiologia, Hospital Universitário Walter Cantídio, Universidade Federal do Ceara, Fortaleza, Brazil; ^3^ Department of Radiology, Ribeirao Preto Medical School, University of São Paulo, Ribeirao Preto, Brazil; ^4^ Unidade de Pesquisa Clínica, Hospital Universitário Walter Cantídio, Universidade Federal do Ceara, Fortaleza, Brazil

**Keywords:** postsurgical hypoparathyroidism, parathyroid-related disorders, vertebral fracture, morphometric fracture, radiography

## Abstract

**Introduction:**

Hypoparathyroidism (HP) is a rare endocrine disease and there are little data available on the risk of fragility fractures in these patients. PTH deficiency results in a positive bone balance with higher bone mass in all skeletal sites. However, whether these structural and dynamic skeletal changes have a negative impact on the fracture risk, it is not known.

**Methods:**

Aiming to investigate the risk of insufficiency vertebral fractures in HP, defined using morphometric criteria, a consecutive sampling of 44 women with chronic postsurgical HP was compared to a control group of 44 adult healthy women, matched by age with patients. Vertebral fractures were analyzed by the semiquantitative Genant’s method followed by quantitative vertebral morphometry.

**Results:**

Morphometric vertebral fractures were identified in 5/44 (11.4%) patients and in 3/44 (6.8%) controls (p=0.731). Most fractures were classified as Genant II and III grades in HP patients, whereas most were Genant I in controls. A logistic regression multivariate analysis was conducted in which age, BMI and parathyroid status were the independent variables, and morphometric vertebral fracture was the dependent variable, but none of these factors was a significant predictor of fracture in this population (OR 1.01, 95% CI 0.96-1.07, p=0.634 for age; OR 2.24, 95%CI 0.47-10.50, p=0.306 for the presence/absence of HP and OR 0.92, 95% CI 0.76-1.10, p=0.369 for BMI).

**Conclusion:**

The results of this study cannot ensure a higher risk of fragility vertebral fractures in postsurgical HP patients. Instead, we only observed higher Genant grade classification of the deformed vertebrae in our sample.

## 1 Introduction

Hypoparathyroidism (HP) is an endocrine disorder characterized by insufficient production of parathyroid hormone (PTH) resulting in derangements of mineral homeostasis, including hypocalcemia and hyperphosphatemia. It’s most often caused by accidental damage or removal of parathyroid glands in neck surgery (75% of all cases) (1).

Its prevalence has been estimated in 37 per 100,000 person-years in the United States, setting a total of approximately 115,000 cases and being categorized as an orphan disease in the aforementioned country (2). A very low prevalence of postsurgical HP was also assessed in Denmark, where 22 cases per 100,000 person-years was estimated (3). However, the risk of this disease is heavily dependent on surgical expertise and may be higher in centers where the experience is not as high.

Although neuromuscular symptoms are often the initial clinical manifestation of HP, this disorder can also affect the function of many organ systems causing neuropsychological and neurological dysfunction, soft tissue calcifications, typically seen on the brain (basal ganglia) and kidney, and skeletal disturbances represented by a dense bone with low remodeling (3–6). Nevertheless, the higher bone mineral density (BMD) might not translate into higher skeletal strength.

It’s known that the standard treatment, consisting of oral calcium and active vitamin D analogs, does not correct all the clinical and metabolic disturbances of HP, and the high doses required may contribute to unwanted complications such as extra skeletal calcifications and impaired renal function (1). Therefore, replacement therapy with recombinant human intact PTH, rhPTH (1–84), is an attractive option, recently approved by the FDA for patients not well controlled with standard treatment. However, skeletal effects of this therapy have not been fully characterized and prospective data on the risk of fracture with rhPTH (1–84) are not available (6, 7). Similarly, fracture data in HP patients on standard treatment are scarce in the literature (5, 8, 9).

In order to provide further knowledge on the risk of fractures in HP, a cross-sectional study was performed, aiming to investigate the risk of morphometric vertebral and clinical fractures related to HP.

## 2 Materials and methods

### 2.1 Subjects

The study group included patients with established diagnosis of chronic postsurgical HP followed at the metabolic bone diseases outpatient clinic, in which all the HP patients from the institution are registered. These patients have been regularly followed since May 2007. This was a consecutive sampling in which every woman with chronic HP, previously submitted to thyroidectomy due to non-malignant causes, was invited to participate during the period of January to December 2018. Chronic HP was defined as hypocalcemia with inappropriately low PTH level, requiring calcium and/or vitamin D analogs supplementation for more than 6 months. This therapy was adjusted to maintain serum calcium concentrations slightly below normal (up until 0.5 mg/dL below normal) or in low normal range, that is from 7.8 to 8.5 mg/dL, to maintain normal serum phosphate, calcium-phosphate product below 55 mg²/dL², urinary calcium below 300 mg/24 h and to avoid symptoms of hypocalcemia.

The control group consisted of adult women selected in a consecutive sampling from the university hospital workers, matched by age (± 2 years) with patients (1:1). Both patients and control groups consisted of a brown-skinned color population, resulting from extensive admixture among three ancestral roots (Europeans, Africans and Amerindians), characteristic of the state of Ceara, where the representation of black people is very small. However, since some patients were elderly, we could not assemble the entire control group among the hospital staff. Therefore, it was necessary to select part of the controls from the general endocrinology outpatient clinic, in the same institution, stressing that all of them were submitted to the same exclusion criteria as patients. They were also selected in a consecutive sampling technique and matched by age (± 2 years) with HP patients (1:1).

The exclusion criteria for both patients and controls were the diagnosis of thyroid cancer or other malignancies, personal history of metabolic bone diseases other than osteoporosis, premature ovarian failure, hepatic disease, chronic renal failure, neurological diseases, alcohol abuse and use of medications known to interfere with mineral metabolism (glucocorticoids and anticonvulsants). Chronic renal failure was defined as plasma creatinine above 1.5 mg/dL.

This study was approved by the Ethics Committee of the Universidade Federal do Ceara and informed written consent was obtained from all patients and controls.

### 2.2 Methods

#### 2.2.1 Clinical and biochemical aspects

An interview-based questionnaire aiming to obtain relevant information about bone health was prospectively applied to patients and controls. The questions evaluated age at menopause, menstrual disorders, history of clinical fractures, site, mechanism and date of the reported fractures, history of backache, alcoholism, smoking, comorbidities, all medicines regularly used by each subject as well as the current or past use of antiosteoporotic drugs and hormonal therapy.

Demographic, clinical, and laboratorial data of the patients were retrospectively assessed from their individual hospital charts. Laboratory tests during the follow-up are also registered in a data collection sheet (an outpatient follow-up record) covering the most important data for each patient. Laboratory tests of a fraction of the control group were prospectively assessed.

Serum concentrations of total calcium, phosphorus, magnesium, alkaline phosphatase, creatinine and albumin, as well as urinary calcium, were determined on an automatic biochemical analyzer (CMD 800ix1, Wiener Lab Group, Argentina) on the day of the blood collection. Calcium, phosphate, magnesium and alkaline phosphatase reference ranges were 8.3-10.5 mg/dL, 2.5-5.6 mg/dL, 1.7-2.5 mg/dL and 45-250 U/L, respectively. Calcium was adjusted for albumin concentration (10). Serum TSH and “intact” PTH concentrations were measured by chemiluminescence (IMMULITE 2000, Siemens, Llanberis, United Kingdom); PTH normal range 11-67 pg/mL. intra-assay and inter-assay coefficients of variation are 5.7% and 8.8%, respectively; TSH normal range 0.5-4.8 uU/mL, intra-assay and inter-assay coefficients of variation are 3.9% and 4.8%, respectively. Serum 25OHD was determined by chemiluminescence (Architect, Abbott, Sligo, Ireland), with intra-assay and inter-assay coefficients of variation 2.9 and 5.5%.

#### 2.2.2 Vertebral morphometry

Each subject underwent thoracic and lumbar spine radiographs in the anteroposterior and lateral views in breath holding after expiration, in the fasting state. All films were taken at a focus-film distance of 100 cm and the X-ray beam was centered at T8 and L3 for the thoracic and lumbar spine, respectively. Radiographs were analyzed in blind assessment by two independent radiologists using the semiquantitative Genant’s method, according to which vertebrae T4-L4 were graded on visual inspection and without measurements as normal (grade 0), mildly deformed (grade 1, approximately 20-25% reduction in anterior, middle and/or posterior height and a reduction area of 10-20%), moderately deformed (grade 2, approximately 25-40% reduction in any height and a reduction in area of 20-40%) or severely deformed (grade 3, approximately 40% reduction in any height and area) (11). Deformed vertebrae were submitted to a quantitative assessment in which six points per vertebra were marked, positioned on the outer edge of the upper and lower endplates, defining the anterior, posterior, and middle vertebral heights in millimeters. A vertebral deformity was defined by the demonstration of more than 20% reduction between two of these measures in the same vertebra or a 20% reduction between any one of these measures and the measurement of the corresponding height of the adjacent vertebral body. All images in which vertebral deformities were identified as well as the difficult and doubtful cases were re-examined by a senior radiologist with high specific expertise in musculoskeletal images. Patients with unreadable films were excluded.

#### 2.2.3 Statistical analysis

Data are presented as mean and standard deviation (SD), as well as median and range, and frequencies in percentages. Between-group differences were analyzed, as appropriate, using Fisher’s exact test and Pearson qui-squared test for categorical variables, and two-sample t-test for continuous variables, after testing for normal distribution. The relative risk for morphometric vertebral fracture was calculated in a multivariate logistic regression model. A p-value less than 0.5 was considered significant. All calculations were performed using the Statistical Package for Social Sciences (SPSS 20.0) for windows.

## 3 Results

### 3.1 Clinical and biochemical characteristics of HP patients

From May 2007 through December 2018, 179 patients with established diagnosis of chronic HP were registered at the metabolic bone diseases clinic in the institution. However, 11 patients manifested late recovery of parathyroid function and discontinued calcium and vitamin D analogs supplementation, and 39 patients did not attend medical visit in the previous year (loss of follow-up). Therefore, a total of 129 chronic HP patients (90% women) were in active medical follow-up. HP was due to neck surgery in 124/129 (96.1%), congenital in 4/129 (3.1%) and idiopathic in 1/129 patient (0.8%). Concerning postsurgical cases, 53.2% (66/124) had neck surgery performed due to malignant diseases and 46.8% (58/124) due to benign thyroid diseases.

Among the 58 patients with chronic postsurgical HP due to benign thyroid diseases, 7 met exclusion criteria. The reasons for exclusion were glucocorticoid treatment for rheumatoid arthritis, systemic lupus erythematous or Addison’s disease (n=3), chronic renal failure and stroke (n=1), myelodysplastic syndrome (n=1), type 1 neurofibromatosis (n=1) and male gender (n=1). Therefore, 51 women were invited to participate in this study. However, 7 of them did not attend the radiographic test or declined to participate and were excluded from this analysis.

The analysis of the results was performed in the final sample of 44 women diagnosed with chronic postsurgical HP who had been operated on for benign thyroid diseases. The median duration of postsurgical HP was 12.1 years and ranged from 1 to 52 years (16.0 ± 12.7 years). Medical indications for surgery were nontoxic nodular disease in 40/44 (90.9%) patients and hyperthyroidism in 4/44 (9.1%). The mean age of the 44 included patients was 58.7 ± 13.2 years (range 29.7-81.3 years) and 34/44 (77.3%) were older than 50 years old. The minimum serum concentration of total calcium measured in these patients during the outpatient medical visits was 6.4 ± 1.1 mg/dL (range 3.5-7.8 mg/dL) and concomitant intact PTH concentration was 6.2 ± 4.9 pg/mL (range 0.6-18.1 pg/mL). Maximum serum phosphate was 6.1 ± 1.1 mg/dL (range 4.5-8.6 pg/mL). All patients were on levothyroxine replacement therapy (109 ± 29 mcg/day) and the media of the last three serum TSH concentrations was 4.6 ± 9.1 uU/mL (median 2.5, range 0.02-55.8 uU/mL). Only two patients (4.5%) had below normal TSH concentrations in this analysis.

In the last medical visit, calcium supplementation and calcitriol were required by 100% of the patients. The amounts of supplemental calcium and calcitriol required were 2,100 ± 1,000 mg/d (range 600-3,600 mg/d) and 0.6 ± 0.3 mcg/d (range 0.25-1.5 mcg/d), respectively. Thiazide diuretics were required by 14/44 (31.8%) patients for controlling hypercalciuria in the dosage of 30.8 ± 11 mg/d of hydrochlorothiazide (range 25-50 mg/d). **Table 1** shows the mean biochemical parameters of the 44 chronic HP women in the last medical visit. Although the mean albumin-adjusted total calcium concentration was within the reference range, only 20/44 (45.4%) were within the therapeutic target for HP patients (7.8-8.5 mg/dL); 12/44 (27.3%) had albumin-adjusted total calcium < 7.8 mg/dL and 12/44 (27.3%) had albumin-adjusted total calcium > 8.5 mg/dL. By contrast, most patients, 35/44 (79.5%), had normal serum phosphate (≤ 5.6 mg/dL); only 9/44 (20.5%) had high concentrations. The calcium-phosphate product was ≤ 55 mg²/dL² in 40/44 patients (90.9%). Concerning urinary calcium excretion, 14/42 (33%) subjects had urinary calcium between 2 and 4 mg/Kg/d, 16/42 (38%) had low urinary calcium excretion (<2 mg/Kg/d) and 12/42 (29%) had hypercalciuria (above 4 mg/Kg/24 h). During the complete period of follow-up, 8/44 (18.2%) patients had at least one episode of hypercalcemia (> 10.5 mg/dL), setting the diagnosis of vitamin D intoxication.

**Table 1:** Biochemical evaluation of the patients with postsurgical hypoparathyroidism on standard treatment with calcium and calcitriol and controls.

	HP patients		Controls	
Serum calcium (mg/dL)	8.4 ± 0.7	8.4 (6.9-9.9)	9.5 ± 0.4	9.6 (8.7-9.9)
Albumin (g/dL)	4.5 ± 0.4	4.4 (3.9-5.5)	4.2 ± 0.3	4.2 (3.8-4.8)
Albumin-adjusted total calcium	8.1 ± 0.7	8.2 (6.7-10)	9.2 ± 0.6	9.4 (8.3-10.0)
Serum phosphate (mg/dL)	5.0 ± 0.8	4.9 (3.5-7.2)	3.7 ± 0.6	3.7 (2.7-4.5)
Alkaline phosphatase (U/L)	125 ± 43	120 (72-235)	158 ± 41	177 (65-201)
Calcium-phosphate product (mg^2/^dL^2^)	42.0 ± 7.4	42.0 (29.4-55.5)	–	–
Urinary calcium excretion (mg/d)	177 ± 123	152 (11-474)	153 ± 47	156 (85-240)
Urinary calcium excretion (mg/Kg/d)	2.7 ± 1.7	2.3 (0.2-6.3)	2.7 ± 1.1	2.5 (1.3-5.1)
Serum magnesium (mg/dL)	1.9 ± 0.3	1.9 (1.3-2.2)	1.8 ± 0.2	1.9 (1.3-2.1)
Creatinine (mg/dL)	0.8 ± 0.3	0.8 (0.5-1.5)	0.9 ± 0.3	0.9 (0.6-1.5)
25OHD (ng/mL)	36.9 ± 10.1	34.1 (17.7-68.3)	–	–
TSH (uU/mL)	4.6 ± 9.1	2.5 (0.02-55.8)	1.7 ± 1.3	1.1 (0.7-5.2)

Values expressed as media ± SD and median (minimum-maximum).

### 3.2 Fracture risk analysis

The group of 44 women with chronic postsurgical HP was compared to a control group composed of 44 women aged 57.8 ± 13.6 years, being 24 selected from the hospital staff and 20 selected from the general endocrinology outpatient clinic. The controls selected from the clinics were referred to medical care due to nontoxic thyroid nodule (n=6), hypothyroidism (n=6), overweight (n=3), pre-diabetes (n=2), type 2 diabetes (n=2) and osteoporosis (n=3). **Table 2** shows the basal clinical characteristics of patients and controls. Most subjects (34/44; 77.3%) were older than 50 years old. It’s noteworthy that, since measurement of bone mineral density by DXA (dual energy X-ray absorptiometry) was not included in our protocol, the diagnosis of osteoporosis was self-reported by the subjects or, alternatively, the diagnosis of osteoporosis was established after the occurrence of a fragility fracture and exclusion of secondary causes. The only antiosteoporotic medication used for patients and controls was the oral bisphosphonate alendronate, since this was the only one provided by the public health care system in our region, and no patient and control had current or past use of estrogens and/or progestins.

**Table 2:** Baseline clinical characteristics of patients with postsurgical hypoparathyroidism and controls.

	HP patients (n=44)	Controls (n=44)	Statistics p-value
Age (yr)	58.7 ± 13.2	57.8 ± 13.6	0.770^a^
Height (cm)	155 ± 7	158 ± 7	0.051^a^
BMI (Kg/m²)	27.6 ± 5.1	25.6 ± 3.8	0.056^a^
Tabagism n (%)	3 (6.8%)	2 (4.5%)	> 0.999^b^
Hypothyroidism n (%)	44 (100%)	6 (13.6%)	< 0.001^b^
Osteoporosis n (%)	6 (13.6%)	6 (13.6%)	> 0.999^b^
Osteoporosis treatment* n (%)	5 (11.3%)	4 (9.1%)	> 0.999^b^
Diabetes n (%)	6 (13.6%)	4 (9.1%)	0.738^b^
Hypertension n (%)	16 (36.4%)	14 (31.8%)	0.652^c^

^a^Student’s T test, ^b^Fisher’s exact test, ^c^Pearson qui-squared test. *Alendronate

#### 3.2.1 Morphometric vertebral fractures

Morphometric vertebral fractures were identified in 5/44 (11.4%) postsurgical HP patients, who had 6 fractures, and in 3/44 (6.8%) controls, who had 4 fractures (p=0.731). Most were classified as Genant II and III grades in HP patients, whereas most were Genant I in the controls (**Table 3**).

**Table 3:** Site and Genant classification of morphometric vertebral fractures in patients with postsurgical hypoparathyroidism and controls.

Subject	Age at HP diagnosis (yr)	Age at radiography (yr)	Time since diagnosis (yr)	Fracture bone site	Genant grade
P1	31	78	47	L3	III
P2	48	58	10	T11	I
P3	16	30	14	T6, T10	II, I
P4	56	66	10	L3	II
P5	57	80	23	T6	III
C1	–	63		T4, T5	I, I
C2	–	47		T7	I
C3	–	71		T6	II

P1-P5, patients; C1-C3, controls.

Among the HP patients with morphometric vertebral fractures, one of them was a 78-year-old woman submitted to cervical surgery due to Graves’ disease. Considering that previous hyperthyroidism could be a predisposing factor for fractures, when we excluded the four HP patients with previous hyperthyroidism, as well as their age-matched controls, from the analysis, vertebral fractures were present in 4/40 (10%) HP patients and 2/40 (5%) controls (p=0.676). The risk of morphometric vertebral fractures in HP patients was not different when patients with previous hyperthyroidism were excluded from the analysis.

Considering that the control group was selected from two different scenarios (since, for the older patients, age-matched controls couldn’t be selected among the hospital workers), other two subgroup analysis were performed. Analyzing only the subgroup of 24 HP patients (age 50.1 ± 10.2 years) whose controls were selected among the hospital workers, morphometric vertebral fractures were identified in 1/24 patients (4.2%) and 2/24 (8.3%) controls (p>0.999). When we analyzed the subgroup of 20 HP patients (age 68.9 ± 8.2 years) whose controls were selected from the general endocrinology outpatient clinic, morphometric vertebral fractures were identified in 4/20 patients (20%) and 1/20 controls (5.0%) (p=0.341). Age-matched controls could not be selected among workers because most people were retired at this age.

Considering that fragility fractures are more frequent in postmenopausal women, we performed another subgroup analysis including only HP patients aged 50 years old and over. This group was composed of 34 HP patients (age 64.2 ± 8.7 years) and their controls. The morphometric vertebral fracture prevalence rate was 11.8% (4/34) in these HP patients and 5.9% (2/34) in the controls (p=0.673). When we analyzed the subgroup of 10 HP patients under the age of 50 (age 39.7 ± 6.4 years.), morphometric vertebral fractures were found in 1/10 (10%) HP patients and 1/10 (10%) controls (**Table 4**).

**Table 4:** Incidence of morphometric vertebral fractures in subgroups of patients with postsurgical hypoparathyroidism and controls.

	Hypoparathyroidism	Controls	p-value
Complete sample (n=44)	5/44 (11.4%)	3/44 (6.8%)	0.731
Patients without previous hyperthyroidism (n=40)	4/40 (10%)	2/40 (5%)	0.676
Patients, hospital workers control group (n=24)	1/24 (4.2%)	2/24 (8.3%)	> 0.999
Patients, endocrinology clinic control group (n=20)	4/20 (20%)	1/20 (5.0%)	0.341
Patients aged ≥ 50 yr (n=34)	4/34 (11.8%)	2/34 (5.9%)	0.673
Patients aged < 50 yr (n=10)	1/10 (10%)	1/10 (10%)	–

Although we could not demonstrate a higher prevalence of morphometric vertebral fractures in HP patients, we observed that the fractured HP patients had more deformed vertebrae, as assessed by the semiquantitative Genant’s method and quantitative assessment of vertebral heights (**Table 3**). Besides, although non-statistically significant, the subgroup of HP patients whose controls were selected from the general endocrinology outpatient clinic seemed to have a higher proportion of vertebral fractures, being important to point out that these were the oldest patients. Therefore, aiming to test the effect of some potential factors on the risk of morphometric vertebral fractures, we conducted a logistic regression multivariate analysis in which age, BMI and parathyroid status were the independent variables, and morphometric vertebral fracture was the dependent variable. We found that none of these factors was a significant predictor of fracture in this population (OR 1.01, 95% CI 0.96-1.07, p=0.634 for age; OR 2.24, 95%CI 0.47-10.50, p=0.306 for the presence/absence of HP and OR 0.92, 95% CI 0.76-1.10, p=0.369 for BMI).

#### 3.2.2 Clinical fragility fractures

Clinical fragility fractures (**Table 5**) were reported by 5/44 HP patients (11.4%) and 3/44 (6.8%) controls (p=0.731). These five HP patients had 8 clinical fragility fractures, being 3 vertebral, 1 femoral, 1 wrist and 3 in minor sites, whereas the three controls reported 3 fractures in minor bone sites.

**Table 5:** Clinical fragility fractures in patients with postsurgical hypoparathyroidism and controls.

Subject	Age at HP diagnosis (yr)	Age at fracture (yr)	Time to 1^st^ fracture (yr)	Fracture site
P1	31	57 and 69	26	Wrist and vertebrae
P2	63	55	before HP diagnosis	Foot
P3	72	63 and 64	before HP diagnosis	Patella and femur
P4	57	58 and 79	1	Foot and vertebrae
P5	45	53	8	Vertebrae
C1	–	44	–	Patella
C2	–	60	–	Foot
C3	–	67	–	Patella

P1-P5, patients; C1-C3, controls.

## 4 Discussion

Hypoparathyroidism is a rare endocrine disease and there are little data available on the risk of fragility fractures in these patients. Since PTH plays a central role in the regulation of bone remodeling, PTH deficiency decreases bone resorption and formation process, resulting in a positive bone balance with higher bone mass in all skeletal sites (1). However, whether these structural and dynamic skeletal changes have a negative impact on the fracture risk, it is not known. In this study, we did not find a higher risk of fragility fractures in HP patients.

To our knowledge, the first study on HP and fracture risk was published in 1995, in which the incidence of spinal deformity was studied in 33 postmenopausal women who had been submitted to a total thyroidectomy more than three years before, due to differentiated thyroid cancer (12). These patients were divided in two groups according to parathyroid status, being 13 HP patients (age 42-80 years) and 20 patients with normal parathyroid function (age 48-78 years). Thoracic and lumbar spine radiographs obtained at the time of thyroidectomy and after 12 years of follow-up revealed a lower mean incidence of newly deformed spines in HP patients (0.051 ± 0.089 vs. 0.163 ± 0.179/year, p<0.05). Since the rate of BMD loss was significantly lower in HP women in early postmenopausal period, the authors stated that HP provides protection against accelerated bone loss, resulting in reduced fragility of the spinal bones. The number of deformed vertebrae in each patient as well as the grade of deformity were not informed by the authors.

The first evidence of increased risk of fragility fractures in postsurgical HP dates from 2013 (8). In this study, 33 postmenopausal women were assessed for the impact of the HP on the frequency of subclinical vertebral fractures, being 16 women with postsurgical HP, previously submitted to thyroidectomy due to nontoxic benign multinodular goiter, and 17 normal parathyroid controls. Patients and controls were matched by age (62.3 ± 8.9 and 58.0 ± 6.1 years, respectively) and BMI (30.3 ± 4.2 and 28.5 ± 5.5 Kg/m^2^, respectively). The HP patients were on treatment for 15.3 ± 12.4 years (range 0.5-51 years), and the daily intake of elemental calcium and calcitriol was 1,713 ± 419 mg and 0.33 ± 0.18 mcg, respectively. Serum levels of albumin-adjusted total calcium were 2.01 ± 0.26 mmol/L (8.04 ± 1.04 mg/dL). Radiographic evaluation of vertebral morphometry revealed fractures in 10/16 HP patients (63%) and in 2/17 (12%) controls. Most of these patients (6/10) had multiple vertebral fractures, as well as the two fractured controls. One patient had a previous nonvertebral fracture, not reported by any control. The authors concluded that the high frequency of morphometric vertebral fractures in HP women indicates deterioration of bone quality due to the low turnover rate and inefficient repair of bone microdamage. In addition, the thickening of trabecular bone reducing its elastic properties and the ability to absorb energy by deforming, when loaded, could be a contributing factor.

Mendonca et al. (8) found morphometric vertebral fractures in 10/16 (63%) HP women and in 2/17 (12%) controls, whereas, in our study, these fractures were detected in 5/44 (11.4%) HP women and in 3/44 (6.8%) controls. In both studies, Mendonca and ours, patients were submitted to surgery due to benign thyroid disease, had similar duration of HP (15.3 ± 12.4 vs. 16.0 ± 12.7 years, respectively) and were on standard treatment with usual daily doses of supplemental calcium (1,713 ± 419 mg vs. 2,100 ± 1,000 mg, respectively) and calcitriol (0.33 ± 0.18 mcg vs. 0.6 ± 0.3 mcg, respectively). However, Mendonca studied only postmenopausal women (age 62.3 ± 8.9, range 45-77 years) while our sample consisted of pre and postmenopausal women (58.7 ± 13.2 years) with a broader age range (29.7-81.3 years). When we analyzed our subgroup of 34 patients aged 50 years and over (age 64.2 ± 8.7 years) and their controls, the morphometric vertebral fracture prevalence rate was not different to that found in our complete series; 11.8% (4/34) of HP patients and 5.9% (2/34) of the controls had vertebral fractures. In a logistic regression model, we also could not demonstrate that age was a risk factor for these fractures in our population of HP patients.

Concerning the diagnosis of vertebral fractures, in Mendonca’s study (8), digitized spine radiographs were assessed by quantitative vertebral morphometry, whereas, in our study, both the original films and the digitized JPEG images (considered to have satisfactory quality) were analyzed by the semiquantitative Genant’s method, followed by quantitative vertebral morphometry of the deformed vertebrae. It should be mentioned that the spines with vertebral deformities, as well as the difficult and doubtful cases (according to the analysis of either of the two independent radiologists), were re-examined in blind assessment by the same senior radiologist with high specific expertise in musculoskeletal radiology who reviewed the images with abnormal vertebral heights in the Mendonca’s study. This fact strengthens the different results of these studies.

In Mendonca’s study, serum 25OHD in HP patients and controls were 40.3 ± 12.6 ng/mL and 32.8 ± 14.0 ng/mL, respectively. In our study, vitamin D sufficiency was evaluated in HP patients (36.9 ± 10.1 ng/mL) and 93.2% (41/44) had normal concentrations (25OHD > 20 ng/mL), but it was not evaluated in our controls. It is possible that, if we had the 25OHD status in our controls, a higher proportion of insufficient concentrations would have been found, since studies in Brazilian population revealed a high prevalence of hypovitaminosis D even in sunny regions (13). Anyway, we don’t believe that differences in vitamin D status could explain differences in fracture rates between HP patients and controls, since the magnitude of the effect of calcium and vitamin D supplementation for reduction of fracture risk is only modest and better demonstrated in those at risk of these deficiencies (14).

There is no obvious reason to explain the markedly different incidence of vertebral fractures in the two Brazilian studies. The risk of osteoporotic fragility fractures varies significantly around the world (15). Considering that Brazil is a multicultural country, and that fracture incidence may differ according to ethnicity, we argue about different fracture probabilities among different Brazilian regions. However, information on the epidemiology of fractures in Brazil is scarce.

Some regional reports on the incidence of hip fractures in different Brazilian regions have been published. The reported age-adjusted annual incidence rate of hip fractures on people older than 60 years-old, was 20.7 and 8.9/10,000 inhabitants for women and men, respectively, in Sobral (16) and 21.7 and 13.0/10,000 inhabitants for women and men, respectively, in Fortaleza, both cities in Northeast Brazil (17). In Marilia, a city in Southeast Brazil, the age-adjusted incidence rate was 29.4/10,000 inhabitants 60 years-old or more/year in 1994, and 35.8/10,000 inhabitants 60 years-old or more/year in 1995 (18). In Porto Alegre (South Brazil), the age-adjusted incidence rate for hip fracture was 202/100,000 inhabitants for people 50 years-old and older (19).

A higher vertebral fracture risk was also demonstrated in an Indian cohort of 104 patients with idiopathic HP (9). Lateral thoracic and lumbar spine radiographs (in DICOM files) were assessed by quantitative vertebral morphometry as well as by the semiquantitative Genant’s method in 104 idiopathic HP patients (aged 37.2 ± 1.45 years, 56 men, 48 women) and in 65 controls (aged 37.5 ± 1.59 years) obtained among patients’ family members. The duration of disease was 15.1 ± 6.6 years and none of the patients were on steroid therapy, but 51% received anticonvulsants. The authors detected a significantly higher prevalence of vertebral fractures in HP patients (19/104, 18.3%) than in controls (3/64, 4.7%) (OR 4.54, 95% CI 1.28 to 16.04, p=0.01). The potential factors determining susceptibility to vertebral fractures were assessed and, on stepwise regression, the most significant predictor for vertebral fractures was longer usage of anticonvulsants (OR 1.15, 95% CI 1.05-1.25, p=0.03). It’s worth noting that hypocalcemic seizures and anticonvulsants could compromise skeletal health and, curiously, 40% of the fractures occurred in the T5-T8 region, possibly related to violent muscle contractions during seizures. Therefore, the inclusion of patients on anticonvulsant therapy could be an important confounding factor for the analysis of fracture risk related to HP. On a similar regression analysis in females, the most significant predictor for vertebral fractures was the postmenopausal status (OR 20.7, 95% CI 2.2-194.8, p=0.008). The authors stated that there was a higher prevalence of vertebral fractures in idiopathic HP patients, especially in postmenopausal women.

Concerning clinical fragility fractures, the data of hospitalizations due to fractures obtained from the Danish National Patient Registry enabled the study of a cohort of 688 patients with postsurgical HP due to non-malignant diseases (20). For each case, three randomly selected controls matched by age and gender were identified from the general population. The authors did not find an increased risk of any fracture in postsurgical HP patients (median age 49, range 17-87 years) (HR 1.03, 95% CI 0.83-1.29). Rather, patients had a significantly lower risk of fracture on upper extremities (HR 0.69, 95% CI 0.49-0.97). Although this was a large study in number of patients, some important information regarding factors influencing bone health were not available.

By analyzing a cohort of 180 patients with nonsurgical HP (median age 49.7 years) in another Danish investigation, the same authors did not find an overall increased risk of fractures (HR 1.40, 95% CI 0.93-2.11) when these patients were compared to a group of 540 gender and age-matched controls. Nevertheless, they found a significantly higher risk of fracture at the upper extremities (HR 1.93, 95% CI 1.31-2.85) (21). The authors suggested that, since patients with nonsurgical HP have increased risk of seizures, the likelihood of falling may be increased, causing a higher risk of fractures in upper extremities.

In a large-scale assessment of nonsurgical HP using the Korean National Health Information Database, the risk of complications was evaluated in a group of 211 patients and 2075 controls, followed during a midway of 9.5 years (22). The diagnosis of fracture was assessed by the ICD-10 codes registered in the database. The prevalence of any type of fractures was not different between the groups, but the risk of vertebral fracture was two times higher in nonsurgical HP patients than in the controls (HR 2.27, 95% CI 1.09-4.72, p=0.029).

Finally, a recent systematic review and meta-analysis of seven observational studies provided a summary of fracture risk in HP, concluding that HP patients have increased risk of vertebral fractures compared to age- and sex-matched non-HP controls; however, this increased risk was not evident in patients with postsurgical HP, but only in nonsurgical HP (23). The authors hypothesized that this disparity could be due to the longer duration of disease in nonsurgical HP, the use of anticonvulsants, as well as the onset of disease at an early age, at the time of acquisition of peak bone mass (23).

Several studies have investigated structural changes of the skeleton and estimated bone strength in HP using trabecular bone score (TBS) and high resolution peripheral quantitative computed tomography (HRpQTC) (24). Cipriani et al. (25) examined TBS in 52 HP patients due to different etiologies, 62% postsurgical, aged 47.6 ± 12.5 years, 14.7 ± 14.2 years since diagnosis. TBS was normal (1.44 ± 0.12), above the threshold of 1.31, reflecting a fracture-resistant microarchitecture in these HP patients. TBS is a surrogate technique for the assessment of cancellous bone microarchitecture and an independent risk factor for fracture in men and postmenopausal women (26). In turn, the estimation of bone strength by HRpQCT and finite-element analysis (FEA) of the distal radius and tibia had contradictory results in two studies by the same group when HP patients were compared to historical controls (27, 28). This discrepancy was attributed to differences in the historical control groups. Using *in vivo* impact microindentation at anterior tibia for the assessment of bone material strength index (BMSi), Starr et al. (29) found 11% lower results in 17 HP subjects, aged 44.5 ± 12 years, compared to matched healthy controls. However, the relationship between estimates of bone strength by FEA or BMSi and bone fracture behavior is unknown.

Our study has some limitations. About half of the controls were selected among hospital workers. Considering that people who are employed are, on average, healthier than those who are not, rates of fractures could be lower in these controls, resulting in an excess risk in the patients. Nevertheless, the absolute number of fractures was higher in the hospital workers’ control group than in the general endocrinology clinic controls. Additionally, the small sample size may have affected the statistical power of this analysis. We didn’t use vertebral fracture assessment (VFA) to diagnose vertebral fractures and we did not assess bone mineral density in the study participants. Also, the lack of 25OHD levels in the control group is a limitation of this study. The major strength of our study is that all patients with the diagnosis of chronic postsurgical HP regularly followed in a reference center of metabolic bone diseases were invited to participate, minimizing the risk of selection bias.

## Data Availability

The raw data supporting the conclusions of this article will be made available by the authors, without undue reservation.

## 5 Conclusion

In conjunction with current scientific literature, the results of this study cannot ensure a higher risk of fragility vertebral fractures in postsurgical HP patients. Instead, we only observed a higher Genant grade classification of the deformed vertebrae in this sample. Thus, we consider that this question remains unresolved, since our study had some limitations concerning the sample size even though this is a representative sampling of a rare disease.
